# Screening of Natural Products Inhibitors of SARS-CoV-2 Entry

**DOI:** 10.3390/molecules27051743

**Published:** 2022-03-07

**Authors:** Pamela González-Maldonado, Nelson Alvarenga, Alberto Burgos-Edwards, Ma. Eugenia Flores-Giubi, Javier E. Barúa, Ma. Cristina Romero-Rodríguez, Ricardo Soto-Rifo, Fernando Valiente-Echeverría, Patricia Langjahr, Guadalupe Cantero-González, Pablo H. Sotelo

**Affiliations:** 1 Biotechnology Department, Facultad de Ciencias Químicas, Universidad Nacional de Asunción, San Lorenzo 111421, Paraguay; pame.54.pg12@gmail.com (P.G.-M.); plangjahr@qui.una.py (P.L.); guada_cantero@hotmail.com (G.C.-G.); 2 Phytochemistry Department, Facultad de Ciencias Químicas, Universidad Nacional de Asunción, San Lorenzo 111421, Paraguay; nelson@qui.una.py (N.A.); aburgos@qui.una.py (A.B.-E.); 3 Biological Chemistry Department, Facultad de Ciencias Químicas, Universidad Nacional de Asunción, San Lorenzo 111421, Paraguay; floresgiubi@qui.una.py (M.E.F.-G.); javierbarua@qui.una.py (J.E.B.); rromero@qui.una.py (M.C.R.-R.); 4 Laboratory of Molecular and Cellular Virology, Virology Program, Institute of Biomedical Sciences, Faculty of Medicine, Universidad de Chile, Santiago 834100, Chile; rsotorifo@uchile.cl (R.S.-R.); fvaliente@uchile.cl (F.V.-E.)

**Keywords:** SARS-CoV-2, entry inhibitor, natural products, pseudotyped virus

## Abstract

The COVID-19 pandemic has led to the search for new molecules with antiviral activity against SARS-CoV-2. The entry of the virus into the cell is one of the main targets for inhibiting SARS-CoV-2 infection. Natural products are an important source of new therapeutic alternatives against diseases. Pseudotyped viruses allow the study of SARS-CoV-2 viral entry inhibitors, and due to their simplicity, they allow the screening of a large number of antiviral candidates in Biosafety Level 2 facilities. We used pseudotyped HIV-1 with the D614G SARS-CoV-2 spike glycoprotein to test its ability to infect ACE2-expressing HEK 293T cells in the presence of diverse natural products, including 21 plant extracts, 7 essential oils, and 13 compounds from plants and fungi. The 50% cytotoxic concentration (CC_50_) was evaluated using the resazurin method. From these analyses, we determined the inhibitory activity of the extract of *Stachytarpheta cayennensis*, which had a half-maximal inhibitory concentration (IC_50_) of 91.65 µg/mL, a CC_50_ of 693.5 µg/mL, and a selectivity index (SI) of 7.57, indicating its potential use as an inhibitor of SARS-CoV-2 entry. Moreover, our work indicates the usefulness of the pseudotyped-virus system in the screening of SARS-CoV-2 entry inhibitors.

## 1. Introduction

Coronavirus disease 2019 (COVID-19), caused by severe acute respiratory syndrome coronavirus 2 (SARS-CoV-2), has become a global pandemic and is still a major global health problem [1]. SARS-CoV-2 is a beta-coronavirus that emerged in late 2019. It is related to the human pathogens severe acute respiratory syndrome coronavirus-1 (SARS-CoV) and to the Middle East respiratory syndrome coronavirus (MERS-CoV), which previously appeared in 2002 and 2012, respectively [2,3]. During the COVID-19 pandemic, SARS-CoV-2 vaccines have been developed, and millions of people have been immunized [4]. However, COVID-19 continues to be a health problem, and in addition to the use of vaccines, antivirals are necessary for the effective treatment of acute SARS-CoV-2 infections [5]. 

Despite there being several antivirals in different stages of development, there are no approved antivirals that inhibit the entry of the virus into the cell [6,7]. Therefore, new effective entry inhibitors are needed for the better management of COVID-19. Viral entry into the host cell is one of the most critical steps in the viral cycle. This process is mediated by the interaction between the SARS-CoV-2 spike glycoprotein and the cellular receptor angiotensin-converting enzyme 2 (ACE-2) [8]. Thus, antivirals that inhibit the viral entry process have important therapeutic potential [9].

Components of natural origin, such as products derived from plants and microorganisms, can be a source of antiviral compounds. Some natural products have shown activity against other coronaviruses, such as SARS-CoV [10]. Additionally, over 85% of patients infected with SARS-CoV-2 in China have received herbal formulae from traditional Chinese medicine along with conventional treatment [11].

In addition, bioinformatics studies such as molecular docking have identified natural products with potential antiviral activity against SARS-CoV-2 by blocking the interaction between the spike protein and the ACE2 receptor. Some of the compounds identified by this strategy are artemisinin and gamma mangostin [12,13]. It is important to note that experimental validation of these data based on bioinformatics models is necessary.

SARS-CoV-2 is highly infective, generates severe clinical cases, and is classified as an aerosol biosafety level (BSL)-3 pathogen [1]. In this situation, the use of pseudotyped viruses represents a robust, fast, and safe alternative to the evaluation of entry inhibitor antivirals in a BSL-2 environment. Due to its versatility, this system has been used for screening entry inhibitors for other viruses [14]. Pseudotyped viral systems have been a valuable tool during the COVID-19 pandemic, especially for the determination of virus-specific neutralizing antibodies [15]. In addition, using this strategy, already-approved drugs were identified as SARS-CoV-2 entry inhibitors [16].

In this work, we screened a library of 41 natural products, including plant extracts, essential oils, and compounds from plants and microorganisms, using an HIV-1-based SARS-CoV-2 pseudotype to identify products with viral entry inhibitory activity. We found that the extract of *Stachytarpheta cayennensis* and β-caryophyllene were able to inhibit SARS-CoV-2 entry into cells. Moreover, our work indicates the usefulness and practicality of this pseudotyped virus in the screening of SARS-CoV-2 entry inhibitors.

## 2. Results and Discussion

### 2.1. Antiviral Screening

The antiviral activity of 21 plant extracts, 7 essential oils, and 13 compounds against SARS-CoV-2 was analyzed. For this purpose, the maximum non-toxic concentration (MNTC) of the natural products was determined (Table 1, Table 2 and Table 3). Subsequently, the antiviral activity at MNTC was determined using an HIV-derived pseudovirus with the spike protein of the SARS-CoV-2 lineage B.1 carrying the D614G mutation (Table 1, Table 2 and Table 3). An antiviral activity greater than 60% was observed with β-caryophyllene (62.10 ± 10.31) and the extracts of *Stachytarpheta cayennensis* (97.03 ± 0.64) and *Phoradendron liga* (82.58 ± 4.75).

In silico analyses have suggested that some essential oil components may be binders of SARS-CoV-2 spike or ACE2 [17]; for that reason, we decided to evaluate the inhibitory effect of essential oils. However, in our experimental conditions, we did not identify essential oils with antiviral activity against SARS-CoV-2. In contrast, some essential oils were found to increase the infectivity of the pseudotyped virus (Table 2).

**Figure 1:**
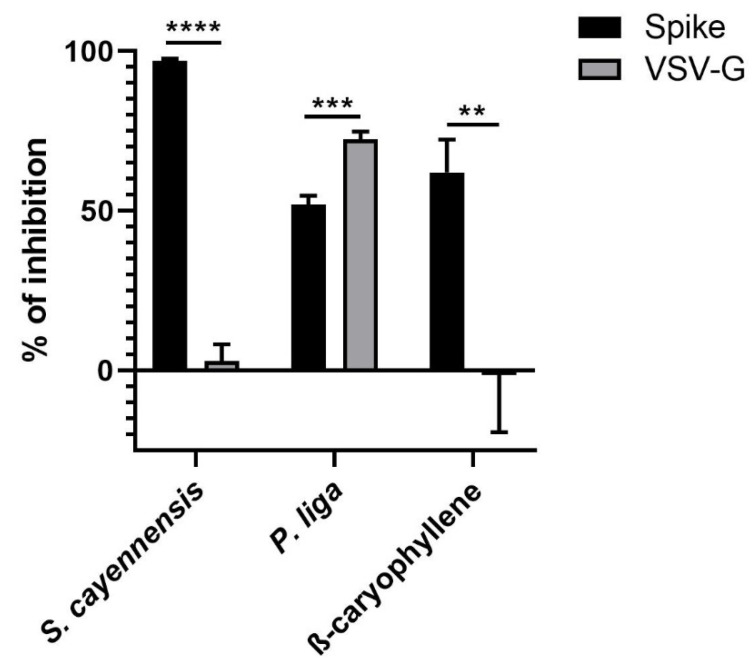
Antiviral activity of natural products against spike- and VSV-G-pseudotyped viruses. HEK-293T ACE2 cells were infected with the corresponding spike- or VSV-G-pseudotyped virus in the presence and absence of different natural products. After 24 h.p.i., the luciferase activity was measured. The % of inhibition was determined as the ratio between treated and untreated cells. ** *p* ≤ 0.01, *** *p* ≤ 0.001, **** *p* ≤ 0.0001.

Since the inhibitory effect of pseudovirus infection can be caused at different stages of infection and not only on the interaction of spike with ACE2, we evaluated the entry inhibition of the best candidates by using a pseudotyped virus carrying the vesicular stomatitis virus glycoprotein G (VSV-G) instead of the spike protein. As shown in Figure 1, *Stachytarpheta cayennensis* extract and β-caryophyllene showed low inhibitory activity against the entry of the VSV-G-pseudotyped virus, showing a specific effect towards the SARS-CoV-2 spike-pseudotyped virus. In the case of *Phoradendron liga*, cytotoxicity was detected in the presence of the VSV-G-pseudotyped virus at 500 µg/mL of the extract; for this reason, the analysis was performed using 250 µg/mL of extract. A greater entry inhibition of the VSV-G-pseudotyped virus (72.46%) compared to the spike-pseudotyped virus (51.89%) was observed, indicating non-specific inhibition.

**Figure 2:**
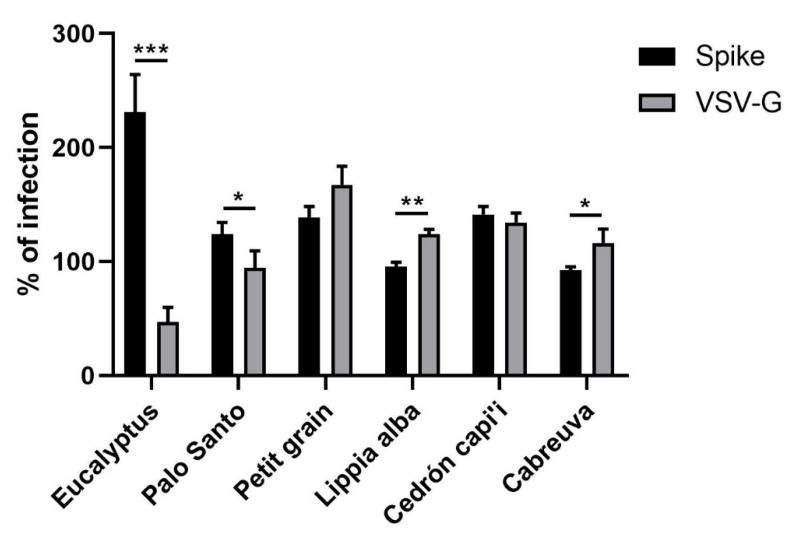
Increased infectivity of pseudotyped virus by essential oils. HEK-293T ACE2 cells were infected with spike- or VSV-G-pseudotyped virus in the presence and absence of essential oils. After 24 h.p.i., luciferase activity was measured. The % of infection was determined as the ratio between the treated and untreated cells. * *p* ≤ 0.05, ** *p* ≤ 0.01, *** *p* ≤ 0.001.

Given that some of the essential oils tested showed an increase in infectivity by the spike-pseudotyped virus, the specificity of infection was also evaluated using the VSV-G-pseudotyped virus. As seen in Figure 2, eucalyptus essential oil increased infectivity only with the spike-pseudotyped virus, indicating that this essential oil could facilitate virus entry mediated by the SARS-CoV-2 spike protein. These results should be confirmed using the whole SARS-CoV-2 virus. Recently, molecular docking analysis has suggested that compounds present in the eucalyptus essential oil could inhibit the SARS-CoV-2 MPro protease. For that reason, it has been proposed as a possible alternative for the treatment of COVID-19 [18]. Our results suggest that caution should be used with the use of eucalyptus essential oil in COVID-19 therapy. 

### 2.2. Cytotoxicity, Inhibitory Concentration, and Selectivity Index

**Figure 3:**
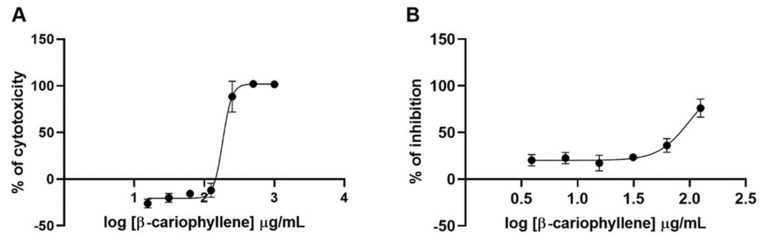
Cytotoxicity and antiviral activity of β-caryophyllene. (**A**) HEK-293T ACE2 cells were treated with increasing concentrations of the compound. After 24 h.p.i., the cytotoxicity was measured by the resazurin method. (**B**) HEK-293TACE2 cells were infected with spike-pseudotyped virus and treated with increasing concentrations of the compound. After 24 h.p.i., the luciferase production activity was measured.

**Figure 4:**
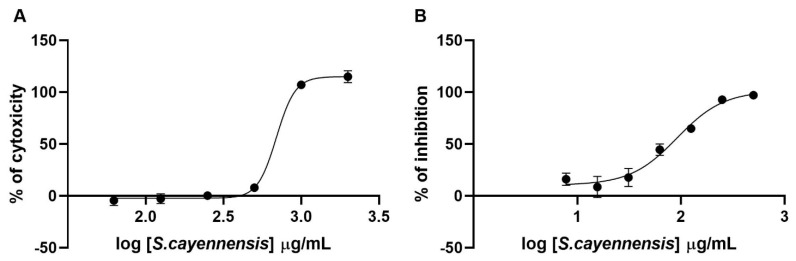
Cytotoxicity and antiviral activity of *Stachytarpheta cayennensis*. (**A**) HEK-293T ACE2 cells were treated with increasing concentrations of the extract. After 24 h.p.i., the cytotoxicity was measured by the resazurin method. (**B**) HEK-293T ACE2 cells were infected with spike-pseudotyped virus and treated with increasing concentrations of the extract. After 24 h.p.i., the luciferase production activity was measured.

β-Caryophyllene showed a 50% cytotoxic concentration (CC_50_) of 183.2 and a 50% inhibitory concentration (IC_50_) of 98.5, with a selectivity index (SI) of 1.85 (Figure 3), while for the methanolic extract of *Stachytarpheta cayennensis*, the CC_50_, IC_50_, and SI were 693.5, 91.65, and 7.57, respectively (Figure 4). 

Antiviral activity of β-caryophyllene has been identified against different enveloped viruses, such as the herpes simplex virus, Newcastle disease virus, and avian infectious bronchitis virus (a gammacoronavirus) [19,20,21]. In addition, a mixture of compounds that includes β-caryophyllene has shown antiviral activity against the alphacoronavirus human coronavirus E229 [22]. In addition, it has been proposed that β-caryophyllene could have anti-inflammatory, antioxidant, and other beneficial effects related to COVID-19 complications [23]. Molecular docking studies have indicated that β-caryophyllene could inhibit different SARS-CoV-2 enzymes, such as SARS-CoV-2 endoribonucleoase, SARS-CoV-2 ADP-ribose-1-phosphatase, SARS-CoV-2 RNA-dependent RNA polymerase, and SARS-CoV-2 MPro protease [17,24]. Furthermore, using this bioinformatic strategy, it has been proposed that β-caryophyllene is capable of binding SARS-CoV-2 spike and ACE2 [17]. Our work is the first report on in vitro assays that identify β-caryophyllene as an inhibitor of SARS-CoV-2.

Members of the genus Stachytarpheta have been associated with different biological activities, such as analgesic, hypotensive, antacid, antihelminthic, diuretic, laxative, lactagogue, purgative, sedative, spasmogenic, vasodilator, vulnerary, and vermifuge properties [25]. Recently, the popular use of *Stachytarpheta jamaicensis* (L.) Vahl and *Stachytarpheta cayennensis* (Rich.) Vahl has been reported for the treatment of COVID-19 in Jamaica [26]. Notably, both species showed low toxicity in laboratory animals [25,27]. Furthermore, *Stachytarpheta cayennensis* and *Stachytarpheta jamaicensis* have been used in traditional medicine to treat inflammation, pain, and fever [25]. The anti-inflammatory activity of both species has been identified in rat and mouse inflammation models [28,29]. Recent therapeutic approaches for COVID-19 patients recommend the use of anti-inflammatories in combination with antivirals [30]. Since *Stachytarpheta cayennensis* extract has anti-inflammatory activity in addition to antiviral activity, it could also be used in the inflammatory stages of COVID-19. In conclusion, our data, together with the aforementioned results, indicate that the *Stachytarpheta cayennensis* extract has potential as an inhibitor of SARS-CoV-2 entry and as a therapeutic agent for COVID-19. However, clinical studies are needed to validate its therapeutic use.

Although there are not many reports of the chemical composition *of Stachytarpheta cayennensis*, *Stachytarpheta jamaicensis* has been reported to be rich in secondary metabolites, showing the presence of several major groups of secondary metabolites, such as alkaloids, flavonoids, phenols, steroids, and terpenoids [25]. Coumarins, flavonoids, tannins, and saponins have been reported among the phenolic compounds present [25]. However, β-caryophyllene, thymol, citral, 1,8-cineole, carvone, and limonene were identified in the essential oil of *Stachytarpheta cayennensis*. Although we identified the SARS-CoV-2 entry inhibitory activity of β-caryophyllene, it is not primarily responsible for the effect observed with the *Stachytarpheta cayennensis* extract, since the extract presented greater activity than β-caryophyllene. It is important to note that there may be other compounds that act synergistically with β-caryophyllene. Molecular docking studies have indicated that thymol, carvone, and limonene can interact with ACE2 and SARS-CoV-2 spike [17]. However, the inhibitory activity of these compounds needs to be confirmed by in vitro or in vivo assays. It is noteworthy that we did not observe inhibitory activity for limonene (Table 3). 

Limited reports are available on the use of pseudotyped viruses for the screening of natural products that inhibit SARS-CoV-2 entry. At the beginning of the pandemic, pseudotyped viruses with the spike protein of MERS and SARS-CoV were used to search for compounds that could inhibit SARS-CoV-2 [31]. Recently, it was reported that the extract of *Punella vulgaris* inhibited the entry of SARS-CoV-2 when using a pseudotyped virus with SARS-CoV-2 spike [32], similar to the system used in this work. Our study also contributes to demonstrating the potential of pseudotyped viruses as a tool for the screening of a large number of natural products that inhibit the entry of SARS-CoV-2 into the cell.

## 3. Materials and Methods

### 3.1. Cell Cultures and Plasmids

HEK293T and stable HEK293T-ACE2-expressing cells [15] were maintained in Dulbecco’s Modified Eagle Medium (DMEM) (Gibco, Grand Island, NY, USA) supplemented with 10% fetal bovine serum (Gibco), antibiotics (Sigma-Aldrich, Saint Louis, MO, USA), and non-essential amino acids (Gibco) at 37 °C and 5% CO_2_ atmosphere. HEK293-ACE2 cells also contained puromycin at a final concentration of 1 µg/mL. The plasmids used were as follows: pNL4.3-ΔEnv-FLuc, spike-G614-Δ19 [15], and pCMV-VSV-G.

### 3.2. Natural Products 

The natural products to be studied were selected mainly based on availability. In the case of plant extracts, most of them showed antiviral activity against HSV-1 [33,34,35,36,37,38]. On the basis of molecular docking, some compounds present in essential oils (β-caryophyllene, caryophyllene oxide, linalool, trans-anethole, S-limonene, R-limonene, cis-verbenol, and guaiol) have been proposed as binders of SARS-CoV-2 spike and ACE2 [17].

The methanolic plant extracts, essential oils, and compounds (Table 1, Table 2 and Table 3) were diluted in DMSO, except for the compound guaiol, which was diluted in methanol.

### 3.3. Cytotoxicity Assay

The maximum non-toxic concentration was assessed using the resazurin method. Briefly, HEK293T-ACE2 cells were cultured at 1 × 10^4^ cells/well in a 96-well plate in the presence of different concentrations of the natural products. Vehicle (DMSO)-treated cells were used as a control and normalizer. Resazurin was added 48 h later, and after 3 h, cell viability was determined. Absorbance was measured on the MultiskanTM GO (ThermoScientific, Waltham, MA, USA) at 570 and 630 nm. The MNTC is the maximum concentration with a cytotoxicity lower than 10%.

### 3.4. HIV-1-Based SARS-CoV-2 Pseudotyped Particles

Pseudotyped viral particles were generated by cotransfection of pNL4.3-ΔEnv-FLuc and spike-G614-Δ19 plasmids (molar ratio, 3:2). HEK293T cells were transfected using the calcium phosphate method [39]. Briefly, 3.2 × 10^6^ cells in a 10 cm plate were transfected with 20 µg of the plasmid mix, 2.5 M calcium chloride, and HEPES-buffered saline 2X (0.28 M NaCl, 0.05 M HEPES, 1.5 mM Na_2_HPO_4_, pH 7.05). The mixture was incubated for 20 min at room temperature and added to the cells. Sixteen hours after transfection, cells were washed, and fresh medium was added. The pseudotyped virus-containing supernatant was collected 48 h after transfection and centrifuged at 3500 rpm for 5 min at room temperature. Viral stocks were aliquoted and stored at −80 °C. Stocks were diluted, HEK293T and HEK293-ACE2 cells were transduced, and firefly luciferase activity was measured 48 hours later using the Dual-Luciferase Reporter Assay System kit (Promega, Madison, WI, USA) and Fluoroskan FL (Thermo Scientific). HEK293T cells transduced with the pseudotyped particles were used as a negative control.

### 3.5. Antiviral Activity

The assay was performed using the maximum non-toxic concentration (MNTC) of each natural product in HEK293-ACE2 cells. A total of 1 × 10^4^ cells in suspension were added to each well of 96-well plates. The mix (50% natural product dilution and 50% virus dilution) was added to the cells. Firefly luciferase activity was measured 48 h later using Dual-Luciferase Reporter Assay System kit (Promega, Madison, WI, USA) and Fluoroskan FL (Thermo Scientific). HEK293T-ACE2 cells transduced with the pseudotyped virus without any natural products were used as control untreated cells. The following formula was used to calculate the percentage of inhibition: 100 − (RLUs of treated cells/RLUs of control untreated cells) × 100. In all assays, RLUs of untreated pseudotyped-virus-transduced HEK293T-ACE2 cells were at least 10-fold over the RLUs of untransduced cells. The selectivity index (SI) was determined as the ratio of CC_50_ versus IC_50_. 

### 3.6. Statistical Analysis

Data are presented as mean ± standard deviation (SD) of at least 3 independent experiments. Differences within each group were subjected to the *t*-test. Statistically significant differences (* *p* ≤ 0.05, ** *p* ≤ 0.01, *** *p* ≤ 0.001, and **** *p* ≤ 0.001) are indicated by lines between the groups being compared.

## Figures and Tables

**Table 1:** Antiviral activity of methanolic plant extracts.

Voucher	Species	MNTC ^1^ (µg/mL)	Antiviral Activity (% of Inhibition)
R. Degen 4164 (10.24)	*Acacia caven (Molina)*	31.25	44.75 ± 4.32
R. Degen 4061 (14.40)	*Acanthospermum australe*	31.25	−16.06 ± 14.66
R. Degen 4042 (5.45)	*Aloysia gratissima*	62.5	35.60 ± 11.72
R. Degen 4224 (7.89)	*Amphilophium paniculatum*	62.5	−45.29 ± 14.43
R. Degen 4064 (15.83)	*Annona emarginata*	15	59.32 ± 8.20
R. Degen 4079 (4.91)	*Austroeupatorium inulifolium*	125	−69.62 ± 30.92
R. Degen 4272 (6.90)	*Baccharis dracunculifolia*	62.5	50.46 ± 5.87
R. Degen 4291 (5.25)	*Calea uniflora*	62.5	25.93 ± 16.21
R. Degen 4044 (5.23)	*Centratherum punctatum*	7.8	−75.99 ± 5.13
R. Degen 4016 (6.30)	*Chromolaena ivifolia*	31.25	2.75 ± 10.94
R. Degen 4198 (4.89)	*Croton paraguayensis*	31.25	54.14 ± 3.16
R. Degen 4236 (4.51)	*Lessingianthus niederleinii*	31.25	59.49 ± 2.50
R. Degen 4257 (8.76)	*Lippia origanoides*	125	1.77 ± 1.77
R. Degen 4321 (10.05)	*Phoradendron liga*	500	82.58 ± 4.75
R. Degen 4065 (3.07)	*Pluchea sagittalis*	15.62	−6.31 ± 18.04
R. Degen 4039 (4.07)	*Pterocaulom angustifolium*	31.25	1.20 ± 12.44
R. Degen 4065 (9.06)	*Solanum sisymbriifolium*	62.5	29.72 ± 4.61
R. Degen 4032 (6.01)	*Solidago chilensis*	31.25	40.97 ± 1.28
R. Degen 4038 (11.05)	*Stachytarpheta cayennensis*	500	97.03 ± 0.64
R. Degen 4127 (11.46)	*Tessaria dodoneifolia*	31.25	−49.87 ± 13.96
R. Degen 4184 (EK 094 PA)	*Zhathoxylum chilopexone*	31.25	25.39 ± 2.33

^1^ Maximum non-toxic concentration.

**Table 2:** Antiviral activity of essential oils.

Species	Vernacular Name	MNTC ^1^ (µg/mL)	Antiviral Activity (% of Inhibition)
*Gonopterodendron sarmientoi*	Palo Santo	37.5	−24.03 ± 10.37
*Citrus aurantium L. var. amara*	Petit grain	31.25	−38.95 ± 9.50
*Myrocarpus frondosus*	Cabreuva	7.8	7.56 ± 2.87
*Anona emarginata*	Aratiku’i	18.25	1.37 ± 2.25
*Eucalyptus globulus*	Eucalyptus	250	−131.35 ± 32.85
*Lippia alba*	Salvia	31.25	4.34 ± 3.69
*Cymbopogon citratusv*	Cedron capi´i	15.62	−41.47 ± 6.84

^1^ Maximum non-toxic concentration.

**Table 3:** Antiviral activity of compounds.

Compound	MNTC ^1^ (µg/mL)	Antiviral Activity (% of Inhibition)
β-Caryophyllene	125	62.10 ± 10.31
Caryophyllene Oxide	31.25	43.82 ± 6.31
Linalool	125	−9.42 ± 0.25
Trans-anethole	500	5.02 ± 0.71
S-Limonene	31.25	−62.63 ± 22.17
R-Limonene	31.25	−27.90 ± 16.41
cis-Verbenol	250	−171.26 ± 32.54
Guaiol	33.3	−60.20 ± 8.02
Macrophominol	36	−91.39 ± 26.65
Acetylphomolactone	4.5	−0.65 ± 6.16
Botryodiplodin	3	−88.21 ± 12.51
Asperline	7.81	−15.48 ± 8.34
Isoasperline	7.19	−19.82 ± 10.21

^1^ Maximum non-toxic concentration.

## Data Availability

The data presented in this study are available on request from the corresponding author.

## Sample Availability

Samples of the compounds are available on request from the corresponding author.

## References

[1] Hu B., Guo H., Zhou P., Shi Z.L. (2021). Characteristics of SARS-CoV-2 and COVID-19. Nat. Rev. Microbiol..

[2] Drosten C., Günther S., Preiser W., van der Werf S., Brodt H.-R., Becker S., Rabenau H., Panning M., Kolesnikova L., Fouchier R.A.M. (2003). Identification of a Novel Coronavirus in Patients with Severe Acute Respiratory Syndrome. N. Engl. J. Med..

[3] Zaki A.M., van Boheemen S., Bestebroer T.M., Osterhaus A.D.M.E., Fouchier R.A.M. (2012). Isolation of a Novel Coronavirus from a Man with Pneumonia in Saudi Arabia. N. Engl. J. Med..

[4] Kyriakidis N.C., López-Cortés A., González E.V., Grimaldos A.B., Prado E.O. (2021). SARS-CoV-2 vaccines strategies: A comprehensive review of phase 3 candidates. NPJ Vaccines.

[5] Santos I.d.A., Grosche V.R., Bergamini F.R.G., Sabino-Silva R., Jardim A.C.G. (2020). Antivirals Against Coronaviruses: Candidate Drugs for SARS-CoV-2 Treatment?. Front. Microbiol..

[6] Sheahan T.P., Sims A.C., Zhou S., Graham R.L., Hill C.S., Leist S.R., Schäfer A., Dinnon K.H., Montgomery S.A., Agostini M.L. (2020). An orally bioavailable broad-spectrum antiviral inhibits SARS-CoV-2 and multiple endemic, epidemic and bat coronavirus. bioRxiv.

[7] Hoffman R.L., Kania R.S., Brothers M.A., Davies J.F., Ferre R.A., Gajiwala K.S., He M., Hogan R.J., Kozminski K., Li L.Y. (2020). Discovery of Ketone-Based Covalent Inhibitors of Coronavirus 3CL Proteases for the Potential Therapeutic Treatment of COVID-19. J. Med. Chem..

[8] Yan R., Yuanyuan Z., Yaning L., Lu X., Yingying G., Qiang Z. (2020). Structural basis for the recognition of SARS-CoV-2 by full-length human ACE2. Science.

[9] Mazzon M., Marsh M. (2019). Targeting viral entry as a strategy for broad-spectrum antivirals. F1000Research.

[10] Islam M.T., Sarkar C., El-Kersh D.M., Jamaddar S., Uddin S.J., Shilpi J.A., Mubarak M.S. (2020). Natural products and their derivatives against coronavirus: A review of the non-clinical and pre-clinical data. Phyther. Res..

[11] Yang Y., Islam M.S., Wang J., Li Y., Chen X. (2020). Traditional Chinese medicine in the treatment of patients infected with 2019-new coronavirus (SARS-CoV-2): A review and perspective. Int. J. Biol. Sci..

[12] Rolta R., Salaria D., Sharma P.P., Sharma B., Kumar V., Rathi B., Verma M., Sourirajan A., Baumler D.J., Dev K. (2021). Phytocompounds of Rheum emodi, Thymus serpyllum, and Artemisia annua Inhibit Spike Protein of SARS-CoV-2 Binding to ACE2 Receptor: In Silico Approach. Curr. Pharmacol. Reports.

[13] Sumaryada T., Pramudita C.A. (2021). Molecular docking evaluation of some indonesian’s popular herbals for a possible covid-19 treatment. Biointerface Res. Appl. Chem..

[14] Li Q., Liu Q., Huang W., Li X., Wang Y. (2018). Current status on the development of pseudoviruses for enveloped viruses. Rev. Med. Virol..

[15] Beltrán-Pavez C., Riquelme-Barrios S., Oyarzún-Arrau A., Gaete-Argel A., González-Stegmaier R., Cereceda-Solis K., Aguirre A., Travisany D., Palma-Vejares R., Barriga G.P. (2021). Insights into neutralizing antibody responses in individuals exposed to SARS-CoV-2 in Chile. Sci. Adv..

[16] Yang L., Pei R.J., Li H., Ma X.N., Zhou Y., Zhu F.H., He P.I., Tang W., Zhang Y.C., Xiong J. (2021). Identification of SARS-CoV-2 entry inhibitors among already approved drugs. Acta Pharmacol. Sin..

[17] da Silva J.K.R., Figueiredo P.L.B., Byler K.G., Setzer W.N. (2020). Essential oils as antiviral agents. Potential of essential oils to treat sars−cov−2 infection: An in−silico investigation. Int. J. Mol. Sci..

[18] Panikar S., Shoba G., Arun M., Sahayarayan J.J., Usha Raja Nanthini A., Chinnathambi A., Alharbi S.A., Nasif O., Kim H.-J. (2021). Essential oils as an effective alternative for the treatment of COVID-19: Molecular interaction analysis of protease (Mpro) with pharmacokinetics and toxicological properties. J. Infect. Public Health.

[19] Schnitzler P., Astani A., Reichling J. (2011). Screening for antiviral activities of isolated compounds from essential oils. Evid. Based Complement. Altern. Med..

[20] Hassanin O., Abdallah F., A.A.Galal A. (2020). In vitro and in vivo experimental trials to assess the modulatory influence of β-caryophyllene on NDV replication and immunopathogenesis. Comp. Immunol. Microbiol. Infect. Dis..

[21] Drevinskas T., Maruška A., Telksnys L., Hjerten S., Stankevičius M., Lelešius R., Mickienė R., Karpovaitė A., Šalomskas A., Tiso N. (2019). Chromatographic Data Segmentation Method: A Hybrid Analytical Approach for the Investigation of Antiviral Substances in Medicinal Plant Extracts. Anal. Chem..

[22] Chatow L., Nudel A., Nesher I., Hayo Hemo D., Rozenberg P., Voropaev H., Winkler I., Levy R., Kerem Z., Yaniv Z. (2021). In Vitro Evaluation of the Activity of Terpenes and Cannabidiol against Human Coronavirus E229. Life.

[23] Jha N.K., Sharma C., Hashiesh H.M., Arunachalam S., Meeran M.N., Javed H., Patil C.R., Goyal S.N., Ojha S. (2021). β-Caryophyllene, A Natural Dietary CB2 Receptor Selective Cannabinoid can be a Candidate to Target the Trinity of Infection, Immunity, and Inflammation in COVID-19. Front. Pharmacol..

[24] Narkhede R.R., Pise A.V., Cheke R.S., Shinde S.D. (2020). Recognition of Natural Products as Potential Inhibitors of COVID-19 Main Protease (Mpro): In-Silico Evidences. Nat. Products Bioprospect..

[25] Liew P.M., Yong Y.K. (2016). Stachytarpheta jamaicensis (L.) Vahl: From Traditional Usage to Pharmacological Evidence. Evid. Based Complement. Altern. Med..

[26] Pieroni A., Vandebroek I., Prakofjewa J., Bussmann R.W., Paniagua-Zambrana N.Y., Maroyi A., Torri L., Zocchi D.M., Dam A.T.K., Khan S.M. (2020). Taming the pandemic? The importance of homemade plant-based foods and beverages as community responses to COVID-19. J. Ethnobiol. Ethnomed..

[27] Olayode O.A., Daniyan M.O., Olayiwola G. (2020). Biochemical, hematological and histopathological evaluation of the toxicity potential of the leaf extract of Stachytarpheta cayennensis in rats. J. Tradit. Complement. Med..

[28] Okoye T.C., Akah P.A., Ezike A.C., Uzor P.F., Odoh U.E., Igboeme S.O., Onwuka U.B., Okafor S.N. (2014). Immunomodulatory effects of Stachytarpheta cayennensis leaf extract and its synergistic effect with artesunate. BMC Complement. Altern. Med..

[29] Sulaiman M.R., Zakaria Z.A., Chiong H.S., Lai S.K., Israf D.A., Azam Shah T.M. (2009). Antinociceptive and anti-inflammatory effects of stachytarpheta jamaicensis (L.) Vahl (Verbenaceae) in experimental animal models. Med. Princ. Pract..

[30] Dabbish A.M., Yonis N., Salama M., Essa M.M., Qoronfleh M.W. (2021). Inflammatory pathways and potential therapies for COVID-19: A mini review. Eur. J. Inflamm..

[31] Chen C.Z., Xu M., Pradhan M., Gorshkov K., Petersen J.D., Straus M.R., Zhu W., Shinn P., Guo H., Shen M. (2020). Identifying SARS-CoV-2 Entry Inhibitors through Drug Repurposing Screens of SARS-S and MERS-S Pseudotyped Particles. ACS Pharmacol. Transl. Sci..

[32] Ao Z., Chan M., Ouyang M.J., Olukitibi T.A., Mahmoudi M., Kobasa D., Yao X. (2021). Identification and evaluation of the inhibitory effect of prunella vulgaris extract on sars-coronavirus 2 virus entry. PLoS ONE.

[33] Gabaglio S., Alvarenga N., Cantero-González G., Degen R., Ferro E.A., Langjahr P., Chnaiderman J., Sotelo P.H. (2019). A quantitative PCR assay for antiviral activity screening of medicinal plants against Herpes simplex 1. Nat. Prod. Res..

[34] Garcia C.C., Talarico L., Almeida N., Colombres S., Duschatzky C., Damonte E.B. (2003). Virucidal activity of essential oils from aromatic plants of San Luis, Argentina. Phytother. Res..

[35] Garber A., Barnard L., Pickrell C. (2021). Review of Whole Plant Extracts with Activity Against Herpes Simplex Viruses In Vitro and In Vivo. J. Evid. Based Integr. Med..

[36] Sakthiselvan P., Madhumathi R., Meenambiga S.S. (2021). Moronic acid: An antiviral for herpes simplex virus. A Centum of Valuable Plant Bioactives.

[37] Simões C.M.O., Falkenberg M., Mentz L.A., Schenkel E.P., Amoros M., Girre L. (1999). Antiviral activity of South Brazilian medicinal plant extracts. Phytomedicine.

[38] Visintini Jaime M.F., Redko F., Muschietti L.V., Campos R.H., Martino V.S., Cavallaro L. (2013). V In vitro antiviral activity of plant extracts from Asteraceae medicinal plants. Virol. J..

[39] Kingston R.E., Chen C.A., Rose J.K. (2003). Calcium Phosphate Transfection. Curr. Protoc. Mol. Biol..

